# Postoperative proliferative vitreoretinopathy development is linked to vitreal CXCL5 concentrations

**DOI:** 10.1038/s41598-021-03294-9

**Published:** 2021-12-14

**Authors:** Souska Zandi, Isabel B. Pfister, Justus G. Garweg

**Affiliations:** 1 Swiss Eye Institute and Clinic for Vitreoretinal Diseases, Berner Augenklinik am Lindenhofspital, Bern, Switzerland; 2 Department of Ophthalmology, Inselspital, Bern University Hospital, University of Bern, Bern, Switzerland

**Keywords:** Biomarkers, Diagnostic markers, Predictive markers, Prognostic markers

## Abstract

The specific changes linked to de novo development of postoperative PVR have remained elusive and were the object of the underlying study. Vitreous fluid (VF) was obtained at the beginning of vitrectomy from 65 eyes that underwent vitrectomy for primary rhegmatogenous retinal detachment (RRD) without preoperative PVR. Eyes developing postoperative PVR within 6 months after re-attachment surgery were compared to those which did not regarding the preoperative concentrations of 43 cytokines and chemokines in the VF, using multiplex beads analysis. For all comparisons Holm’s correction was applied in order to control for multiple comparisons. Twelve out of 65 eyes (18.5%) developed PVR postoperatively. While 12 of the chemokines and cytokines presented concentration differences on a statistical level of *p* < 0.05 (CXCL5, CCL11, CCL24, CCL26, GM-CSF, IFN-γ, CCL8, CCL7, MIF, MIG/CXCL9, CCL19, and CCL25), CXCL5 was the only cytokine with sufficiently robust difference in its VF concentrations to achieve significance in eyes developing postoperative PVR compared to eyes without PVR. CXCL5 may represent a potent biomarker for the de novo development of postoperative PVR. In line with its pathophysiological role in the development of PVR, it might serve as a basis for the development of urgently needed preventive options.

## Introduction

Proliferative vitreoretinopathy (PVR) may develop as a complication of primary rhegmatogenous retinal detachment (primary RRD) in 10% or more of eyes and is a major cause of poor functional outcomes after primarily successful retinal detachment (RD) surgery^1,2^. The number and size of retinal tears are correlated with the risk of its development. This is linked to an increase of cell-signaling mediators, which are most likely released from the damaged tissue and involved in the regulation of inflammatory processes, breakdown of the blood-retinal barrier, wound healing, and scar formation^3–6^. Dedifferentiated retinal pigment-epithelial (RPE) cells contribute to the formation of contractile PVR membranes, which form in response to these cell-signaling mediators^3,7,8^. Clinically, increased levels of a variety of cytokines, chemokines and growth factors have been reported in the vitreous of eyes with RD^9–15^. Our group revealed differences in vitreal cytokine concentrations between early and advanced primary PVR and reported that CCL19 may represent a biomarker for PVR progression^16^. Vitreal cytokine levels in eyes developing postoperative PVR after uncomplicated RD repair in the absence of primary PVR, however, have not yet been addressed. Therefore, this study aimed to investigate the cytokine concentrations in the vitreous fluid (VF) of eyes without preoperative but postoperatively developing PVR after vitrectomy for primary RRD and to compare them to those from eyes without the development of PVR.

## Patients and methods

### Patients

The investigation was designed as a retrospective study involving a consecutive series of eyes undergoing pars plana vitrectomy and gas filling for the treatment of primary RRD. Eyes with any sign of preoperative PVR were excluded. All surgeries were performed by the same surgeon at the Berner Augenklinik am Lindenhofspital, Bern, Switzerland. Patients with any systemic or ocular comorbidities that may potentially influence ocular cytokine levels were excluded (i.e., patients with diabetes mellitus; known rheumatic and autoimmune diseases; systemic treatments involving corticosteroids or immunomodulatory drugs; vitreous hemorrhaging, uveitis, glaucoma, or any concomitant retinal pathology; or who had undergone any intraocular treatment or surgery within 6 months of the RD diagnosis). If both eyes were affected, only the first operated eye was included.

The study was approved by the Ethics Committee of the University of Bern (KEK no. 152/08) and is fully compliant with the tenets of the Declaration of Helsinki in its latest version. Each participant provided informed written consent to the use of their biological materials and clinical data.

### Patient groups

A total of 65 patients were assigned to group 1 (no pre- or postoperative PVR; n = 53) and group 2 (postoperative PVR, n = 12) regarding whether or not they postoperatively developed PVR within 6 months after the first surgery for primary RRD. Part of the samples from group 1 were already included in a previous publication with a different study question^16^.

### Handling of vitreous fluid samples

Approximately 500 µl of undiluted native VF was collected at the beginning of pars plana vitrectomy. After harvesting, VF was immediately stored at − 20 °C and moved to − 80 °C within 2 months, where it remained until the final analysis, which was performed on all samples simultaneously.

### Cytokine analyses

The samples were analyzed using a multiplex beads system (Bio-Plex 100 Array Reader with Bio-Plex Manager software version 6.1; Bio-Rad, Hercules, CA, USA). With this highly sensitive technique, multiple analytes can be detected in parallel using a single small volume sample. We quantified the concentrations of 43 cytokines in each vitreous sample as previously published^16^. All analytic procedures were performed following the manufacturer’s instructions. In short, magnetic microspheres, tagged with a fluorescent label, were coupled to specific capture antibodies and mixed with samples containing unknown cytokine quantities before introducing biotinylated detection antibodies and Streptavidin R-Phycoerythrin. The mixture was then analyzed by flow cytometry. The two lasers of the instrument identify the microsphere type and quantify the amount of bound target. On each test plate, a duplicate concentration standard was run in parallel for each cytokine. The measurements were performed in a blinded manner by a laboratory technician who was experienced in the execution of this technique.

### Statistical analyses

According to the standard curve, the lower limit of quantitation (LLOQ) of the assay working range was set by the manufacturer to about 1 pg/ml (http://www.biorad.com). The concentrations of several cytokines ranged below the curve fit of these standards (out of range). To avoid a bias that would have been introduced by excluding these values, they were set at half of the lowest quantified level for the particular cytokine in question^17^.

The Shapiro–Wilk test was applied to check for the distribution pattern and revealed that data were not normally distributed. The non-parametric Mann–Whitney U test and the Kruskal–Wallis H test were thus employed for the inter-group comparisons. A *p* value of < 0.05 was considered to be significant. Since multiple comparisons increase the risk of introducing a type I error, we applied the sequentially rejective modification of Bonferroni correction, the Holm correction, to control for type I error, without introducing additional type II error^18,19^. The Holm correction progressively adapts the threshold for rejecting the null hypotheses. As a first step, all *p* values are sorted in order of smallest to largest, and *k* is the number of hypotheses. In a second step, the lowest *p* value is compared to *α*/*k*. If the *p* value is lower, the null hypothesis is rejected and the result is significant. The same procedure is applied at the remaining *k*–1 hypotheses, where the threshold of significance is set at *α*/(*k*-1). This procedure is repeated sequentially for each *p* value, until the selected *p* value is not smaller compared to the sequential threshold. All statistical analyses were performed using R (package FSA, software version 3.4.0).

## Results

Sixty-five eyes of 65 consecutive patients admitted to our clinic for primary RRD met the inclusion criteria. Thereof, 34 eyes (52.3%) were phakic. Since we had demonstrated that the lens status does not influence the cytokine profiles in the VF, phakic and pseudophakic eyes were pooled^20^.

The mean age of the patients was 61.3 ± 13.4 years and 38.5% were female.

Postoperative PVR developed in 18.5% of cases: 53 patients did not develop secondary PVR after vitrectomy (group 1), and 12 patients developed PVR within 6 months of primary vitrectomy (group 2). The corresponding PVR stage was CP, type: focal in ten eyes, and only two eyes presented a PVR stage of CA, type circumferential^21^.

**Figure 1:**
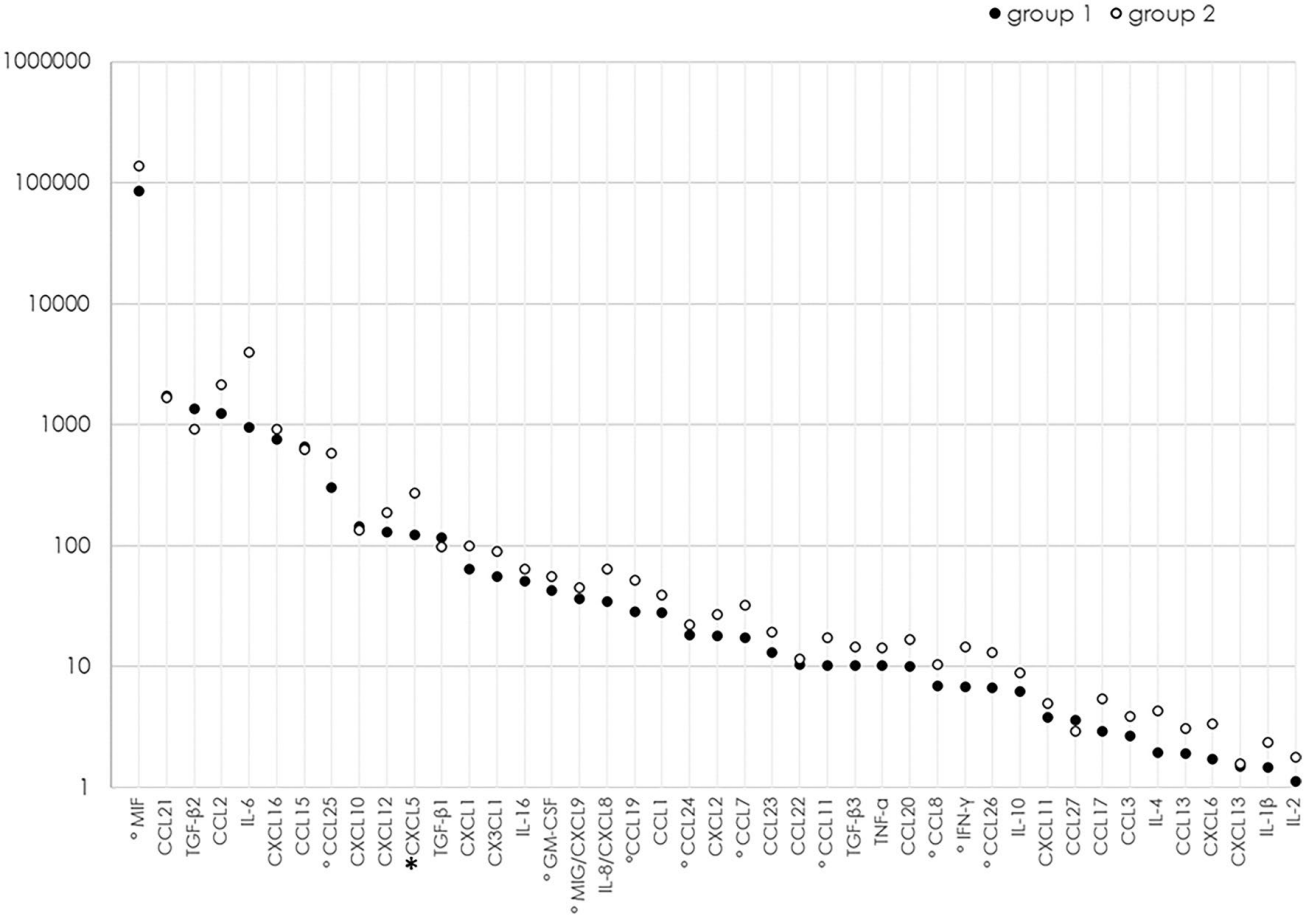
Comparison of vitreal cytokine levels of patients who developed secondary PVR and those who did not on a logarithmic scale. Significant differences of *p* < 0.05 are marked with °, and significant results after the Holm correction are marked with *.

Mean age was similar (group 1: 60.7 ± 14.1 years; group 2: 64.2 ± 9.7, *p* = 0.46), and no difference was observed regarding gender and lens status between the groups (gender: group 1: 34% females; group 2: 58.3% females, *p* = 0.19; lens status: group 1 47.2% pseudophakic; group 2 50% pseudophakic, *p* = 1.0). Both groups were also preoperatively comparable regarding the number of retinal breaks (group 1: 1.6 ± 1.1; group 2: 1.9 ± 1.2, *p* = 0.30) and the extension of RD (group 1: 4.2 ± 1.9; group 2: 4.8 ± 2.8, *p* = 0.42). As outlined in Table 1, the extent of the retinal detachment (≤ 6 clock hours or > 6clock hours), the amount of retinal breaks (≤ 3 retinal breaks or > 3 retinal breaks) and whether the macula was detached (Mac on) or not (Mac off), did not differ in both groups. The concentrations for each of the 43 cytokines in both groups are displayed in Table 2. At a statistical level of *p* < 0.05, an upregulation of 12 cytokines was found in group 2 (CXCL5, CCL11, CCL24, CCL26, GM-CSF, IFN-γ, CCL8, CCL7, MIF, MIG/CXCL9, CCL19, and CCL25). After application of the Holm correction (*p* < 0.00116), the concentration of only one cytokine in the VF, CXCL5, remained significantly higher in eyes that postoperatively developed PVR (group 2; Table 2, Fig. 1).

**Table 1:** Clinical characteristics of RD in group 1 and 2.

	≤ 3 breaks	> 3 breaks	Chi^2^ test
Group 1 n(%)	49 (92.5%)	4 (7.5%)	*p* = 1.0
Group 2 n(%)	11 (91.7%)	1 (8.3%)	

	RD ≤ 6 clock hours	RD > 6 clock hours	Chi^2^ test
Group 1 n(%)	48 (90.6%)	5 (9.4%)	*p* = 0.16
Group 2 n(%)	9 (75%)	3 (25%)	

	Mac off	Mac on	Chi^2^ test
Group 1 n(%)	18 (34%)	35 (66%)	*p* = 0.10
Group 2 n(%)	4 (33.3%)	8 (66.6%)

**Table 2:** Mean concentrations (pg/ml) and standard deviations (SDs), as well as medians and interquartile ranges (IQRs) of cytokines in the vitreous of RRD eyes without PVR and without PVR development after surgical intervention (group 1) and eyes with PVR development after surgery (group 2).

Cytokine	group 1 (n = 53) no postoperative PVR		group 2 (n = 12) postoperative PVR		Mann–Whitney U test (*p* values)
	Mean ± SD (pg/ml)	Median (IQR)	Mean ± SD (pg/ml)	Median (IQR)	
CCL21	1719.9 ± 3921.4	652.8 (400.6–1192.4)	1678.2 ± 1807.6	836.4 (629.0–2454.6)	0.16
CXCL13	1.5 ± 2.7	0.9 (0.7–1.4)	1.6 ± 1.0	1.2 (0.9–2.4)	0.21
CCL27	3.6 ± 9.4	0.6 ( (0–3.5)	2.9 ± 3.3	1.1 (0.1–6.4)	0.53
CXCL5	123.8 ± 147.4	85.7 (8.1–151.1)	273.7 ± 168.2	243.2 (114.3–429.6)	**0.0012**
CCL11	10.2 ± 12.7	5.7 ( (3.9–10.2)	17.3 ± 13.2	12.8 (7.2–26.6)	0.015
CCL24	18.4 ± 22.4	12.8 (8.2–19.3)	22.0 ± 9.4	23.1 (11.9–29.3)	0.02
CCL26	6.7 ± 9.7	4.3 (2.2–6.3)	13.0 ± 12.3	6.9 (4.7–21.0)	0.014
CX3CL1	55.7 ± 65.7	35.0 (18.7–60.9)	89.0 ± 80.8	53.8 (28.4–168.1)	0.14
CXCL6	1.7 ± 2.9	0.4 (0.4–1.6)	3.4 ± 3.7	2.2 (0.4–5.8)	0.053
GM-CSF	42.5 ± 18.4	47.1 (31.3–55.7)	55.8 ± 16.3	55.4 (49.1–64.3)	0.029
CXCL1	63.5 ± 81.7	39.4 (30.1–67.7)	99.7 ± 102.7	63.3 (40.3–85.0)	0.1
CXCL2	18.0 ± 38.8	0.8 (0.8–14.4)	26.9 ± 52.5	0.8 (0.8–35.0)	0.78
CCL1	27.8 ± 44.4	11.4 (7.3–19.9)	38.8 ± 45.9	23.6 (10.4–39.7)	0.072
IFN-γ	6.8 ± 10.3	1.5 (0.8–7.6)	14.6 ± 14.6	9.9 (4.2–27.3)	0.019
IL-1β	1.5 ± 2.7	0.9 (0.4–1.6)	2.4 ± 3.7	0.9 (0.3–2.4)	0.66
IL-2	1.1 ± 1.1	1.1 (0–1.5)	1.8 ± 1.6	1.5 (0.7–1.9)	0.11
IL-4	2.0 ± 4.1	0.1 (0.1–2.8)	4.3 ± 6.3	2.0 (0.1–5.2)	0.1
IL-6	945.8 ± 6283.6	16.1 (5.7–82.8)	3937.0 ± 13,188.5	48.2 (5.5–289.8)	0.43
IL-8/CXCL8	34.5 ± 56.7	15.3 (8.7–35.6)	64.2 ± 92.3	20.3 (11.0–112.0)	0.38
IL-10	6.3 ± 5.4	4.6 (3.3–8.8)	8.8 ± 5.2	7.8 (3.8–14.9)	0.1
IL-16	51.2 ± 38.9	43.0 (21.3–75.2)	64.2 ± 44.9	55.0 (23.5–102.2)	0.31
CXCL10	144.4 ± 263.6	70.0 (40.9–145.2)	134.2 ± 12.5	106.0 (71.8–133.5)	0.19
CXCL11	3.8 ± 5.9	1.7 (0.8–3.5)	4.9 ± 4.8	2.3 (1.7–9.4)	0.11
CCL2	1247.5 ± 1007.7	919.3 (701.1–1515.9)	2153.2 ± 1759.8	1551.4 (749.6–3067.6)	0.11
CCL8	6.9 ± 9.0	4.1 (2.1–7.7)	10.5 ± 7.3	8.5 (5.3–13.4)	0.015
CCL7	17.3 ± 19.8	11.3 (2.6–23.4)	31.9 ± 25.1	26.3 (11.9–49.0)	0.024
CCL13	1.9 ± 2.3	1.3 (0.6–2.3)	3.1 ± 2.3	2.6 (1.1–5.0)	0.063
CCL22	10.4 ± 8.0	10.3 (4.9–13.7)	11.5 ± 9.1	11.5 (1.6–19.4)	0.69
MIF	85,918 ± 81,038	59,913 (23,193–135,776)	136,412 ± 101,434	80,471.0 (54,011–205,000)	0.049
MIG/CXCL9	36.6 ± 85.7	12.9 (7.1–37.3)	45.2 ± 43.6	27.1 (17.4–67.6)	0.019
CCL3	2.7 ± 2.8	1.7 (1.0–3.5)	3.9 ± 3.1	2.8 (2.0–5.5)	0.096
CCL15	654.4 ± 518.8	538.3 (307.9–810.2)	618.6 ± 327.5	540.3 (399.2–761.9)	0.72
CCL20	10.1 ± 18.5	5.13.4–10.0)	16.7 ± 26.8	6.2 (3.2–11.4)	0.53
CCL19	28.3 ± 50.1	11.86.7–24.9)	51.7 ± 50.6	28.5 (15.6–97.1)	0.011
CCL23	13.0 ± 12.6	10.4 (3.8–16.9)	19.4 ± 13.7	17.2 (10.1–23.2)	0.074
CXCL16	757.8 ± 303.9	736.1 (525.7–944.8)	911.8 ± 268.4	900.3 (668.1–1113.9)	0.069
CXCL12	129.8 ± 110.4	84.1 (62.6–148.4)	186.8 ± 136.4	141.9 (76.3–266.7)	0.14
CCL17	2.9 ± 7.0	0.4 (0.4–0.7)	5.4 ± 7.4	0.4 (0.4–11.5)	0.21
CCL25	302.1 ± 380.7	168.1 (73.2–351.3)	579.6 ± 551.6	405.5 (132.4–1217.7)	0.03
TNF-α	10.1 ± 9.6	7.4 (4.4–12.1)	14.2 ± 8.8	11.2 (7.6–20.6)	0.062
TGF-β1	116.0 ± 252.0	2.1 (2.1–109.8)	96.8 ± 239.3	2.1 (2.1–93.7)	0.27
TGF-β2	1357.0 ± 875.2	1182.3 (716.8–1949.5)	913.2 ± 728.7	703.2 (338.6–1260.6)	0.089
TGF-β3	10.2 ± 20.5	1.2 (0.1–9.8)	14.5 ± 39.7	0.1 (0.1–4.5)	0.48

*p* Values which remain significant after Holm correction are marked in bold.

When screening the vitreal cytokines of the 10 smallest versus the 10 largest retinal detachments in both groups (n = 65) no significant difference could be observed after Holms correction was applied, indicating that the extent of the retinal detachment is not the key driver for postoperative PVR development (Table 3).

**Table 3:** Comparison of the vitreal cytokine concentrations between the 10 patients with the smallest vs. biggest extent of retinal detachment (RD).

Cytokine	10 Patients with smallest extent of RD		10 Patients with biggest extent of RD		*p* Value Mann–Whitney U test
	Mean	SD	Mean	SD	
CCL21	915.0	362.5	1893.1	2347.6	0.85
CXCL13	3.2	5.7	1.5	0.9	0.97
CCL27	3.5	4.8	1.8	2.5	0.68
CXCL5	78.2	78.6	148.1	98.0	0.11
CCL11	7.5	5.0	16.1	14.0	0.19
CCL24	22.4	33.2	19.0	10.6	0.32
CCL26	3.5	2.8	9.2	7.6	0.08
CX3CL1	36.3	13.5	83.5	81.3	0.44
CXCL6	0.9	1.2	3.7	4.0	0.06
GM-CSF	41.8	17.7	55.9	15.4	0.14
CXCL1	39.6	17.7	82.9	90.8	0.19
CXCL2	4.4	5.8	36.8	43.0	0.06
CCL1	10.4	5.5	52.5	59.2	0.052
IFN-γ	5.0	6.0	15.8	14.3	0.029
IL-1β	1.1	1.0	2.2	3.8	0.91
IL-2	0.9	0.8	1.8	1.8	0.28
IL-4	1.8	3.8	4.1	7.1	0.44
IL-6	50.9	106.1	4688.0	14450.7	0.32
IL-8 /CXCL8	24.0	21.3	55.8	86.1	0.63
IL-10	5.3	2.5	8.6	4.5	0.17
IL-16	50.8	42.2	72.6	57.8	0.63
CXCL10	96.5	56.5	125.2	146.8	0.80
CXCL11	1.8	1.3	5.3	6.0	0.48
CCL2	959.3	608.6	2152.5	1865.9	0.08
CCL8	5.6	3.9	8.4	8.4	0.85
CCL7	7.3	7.1	34.4	24.6	0.01
CCL13	1.1	0.9	2.9	2.2	0.06
CCL22	13.9	7.5	10.3	8.7	0.32
MIF	90084.6	91024.1	155658.7	100572.4	0.08
MIG/ CXCL9	23.8	26.3	44.5	45.3	0.22
CCL3	2.2	2.2	3.1	2.0	0.14
CCL15	634.3	365.7	662.2	364.6	0.80
CCL20	6.5	4.5	14.4	27.8	0.97
CCL19	16.6	15.2	45.2	52.5	0.19
CCL23	16.8	18.2	17.2	13.8	0.68
CXCL16	774.1	199.2	853.6	365.1	0.91
CXCL12	134.2	58.7	156.1	124.2	0.74
CCL17	0.4	0.1	5.5	7.9	0.22
CCL25	243.6	238.4	647.3	609.0	0.08
TNF-α	7.1	2.4	12.7	8.1	0.22
TGF-β1	110.3	115.2	111.3	257.0	0.25
TGF-β2	1648.8	868.9	1084.9	769.0	0.11
TGF-β3	4.5	5.1	19.6	42.9	0.97

After Holm correction, none of *the p* values remain significant.

## Discussion

The comparison of cytokine profiles in patients with primary RRD without preoperative PVR identified 12 cytokines with increased concentrations in eyes that postoperatively developed PVR, whereas only the chemokine C-X-C motif chemokine ligand 5 (CXCL5) demonstrated a robust upregulation. While this does not exclude a contribution of the remaining 11 biomarkers, it indicates the prominent role of CXCL5 in this biological process. This finding is supported by the fact that when screening the vitreal cytokines regarding the extent of retinal detachment, no significant difference could be observed after Holms correction was applied, indicating that the extent of the retinal detachment is not the key driver for postoperative PVR development. In addition, the amount of retinal breaks and whether the macula was involved or not did not differ between eyes that developed postoperatively PVR to those that did not.

When comparing the cytokine profiles in primary RRD with the ones that underwent vitrectomy for MH and/or ERM^16,20^, more than 30 abundant chemokines/cytokines in the VF were identified, whereas a ≥ tenfold increase in the concentrations was found for 10 cytokines, including CXCL5. After exclusion of any evident PVR before vitrectomy, the cytokinome comparison of eyes postoperatively developing PVR to those without postoperative PVR revealed no predominant role for the other nine previously identified cytokines (CCL26, CCL1, IL-6, CXCL11, CCL7, CCL13, MIG/CXCL9, CCL19 and TGF-β1)^16^.

Contrary to our previous publication, where we compared chemo- and cytokines in patients with and without PVR at primary re-attachment surgery, in the current study we focused only on eyes without any detectable PVR and compared those that developed PVR in the 6 months following RD to those, who did not. While our first study demonstrated the presence of active PVR to be linked to elevated CCL19 concentrations in the vitreous^16^, the underlying study confirms that vitreal CCL19 is upregulated along with eight other cyto- and chemokines. Here, we identified vitreal CXCL5 as the most abundant and thus predictive marker for the de novo development of developing postoperatively PVR.

Our finding of CXCL5 as a key regulator in the development of PVR is pathophysiological well supported by the fact that CXCL5 is implicated in connective tissue remodeling and inflammation^22,23^. Yet, this chemokine also seems to play a role in various acute and chronic, noninfectious inflammatory processes and is involved in host defense and chronic disease progression^24^. CXCL5 is released from endothelial cells^25^ and various inflammatory cells, such as monocytes^22^, and it is involved in recruitment and activation of additional cellular mediators of inflammation, such as macrophages and neutrophils. CXCL5 can bind to CXCR1 and CXCR2 receptors that are predominantly expressed on neutrophils and is activated in response to various triggers including tumor necrosis factor alpha^24,26–28^.

In line with the findings of our paper^16^ Abu-El Asrar et al.^29^ did not find an upregulation of CXCL5 in the VF of eyes with active PVR. Interestingly, Schnyder-Candrian et al.^30^ found that interferon gamma reduces CXCL5 in human monocyte cultures. Limb et al. demonstrated that interferon gamma is upregulated in eyes with PVR. This well explains why CXCL5 is not increased once PVR has been established^31^.

Efforts to reduce the risk of PVR include reduction of surgical trauma, early surgery, pharmaceutical adjuncts, and lower thresholds to use silicone oil or retinotomies, as well as improvements in surgical technique^32^. The identification of a preoperatively present marker of PVR advocates that vitrectomy per se is not a driver of PVR development, which is supported by a study of Joeres et al.^33^, who reported that primary vitrectomy did not reduce the risk of PVR over buckling. Theoretically, the removal of CXCL5 with vitrectomy could have a positive impact on the development of PVR, but such has yet to be demonstrated^34^. That the environmental changes to the cytokinome in response to RD are abandoned by vitrectomy and re-attachment of the retina does not seem likely given the fact that wound healing progresses over months, even after successful RD repair.

That the concentrations of almost all tested chemokines and cytokines were elevated in the vitreous of both primary RRD groups, irrespective of the presence or severity of PVR, may indicate the dimension of the tissue trauma associated with RD^16^. While this does not provide specific clues as to the pathophysiology of PVR induction, it is in agreement with previous studies^2,9,20,35–38^. The direct comparison of the vitreal cytokinome in eyes without postoperative PVR and those newly developing PVR, in contrast, yielded a signal with CXCL5, which we think is specific to the development of PVR, and, as outlined above, well in line with its biological function. Once PVR has established, the drastic changes to the vitreal environment^16^ reduce the relative signal strength of this biomarker compared to several other environmental tissue responses. Once the association of CXCL5 and postoperative PVR development has been confirmed independently, this pathway might be used to control this trigger for the development and progression of PVR. In the absence of supportive results, at the current stage, CXCL5 might be understood as a diagnostic marker for the risk of developing postoperative PVR. However, the cellular source responsible for inducing the increased cytokine concentrations remains to be identified, and their role in the complex pathophysiological process of wound healing in RD has as yet to be determined^39^. RPE and Müller glial cells might be expressing more CXCL5 when injured and when starting the wound healing process, and first attempts to modify the inflammatory environment have already been undertaken^40^. Patients with a higher inflammatory response to RD may be at a higher risk of developing PVR. However, steroids are well capable of controlling inflammation, but after many attempts have not conclusively shown to affect the development of PVR^41–43^.

The main strength of this study is its well-designed selection process with sufficiently large numbers in each group. This allowed us to apply the Holm correction in order to identify the most relevant cytokines amongst the many screened cyto- and chemokines. This is namely important, since the role of cyto- and chemokines in the pathophysiology of PVR has as yet to be established. Replacing the Holm correction by the more conservative Bonferroni correction revealed almost identical results. While the data are consistent, precise, and reliable, the storage conditions must be regarded as a possible weakness of this study. Samples were not immediately stored at −80 °C due to the distance of our operation room to the lab. Principally, a partial degradation of thermosensitive chemokines and cytokines might have taken place, so that the absolute cytokine concentrations have to be carefully weighed, whereas such cannot explain any of the intergroup differences in their concentrations as all samples were treated the same way. Moreover, the cytokine concentrations in the VF reported here and in our previous studies^44–46^ are well in line with published concentrations in the ocular fluids from independent groups^9,12^.

In conclusion, we assessed 43 chemokines and cytokines in retinal detachment without active PVR that later developed PVR and those, which did not, and found increased concentrations in 12 of them. Only one of these, CXCL5, was sufficiently abundant to be unequivocally linked to the de novo development of postoperative PVR as compared to eyes without postoperative PVR.

## Data Availability

Data are available here: https://augenklinik-bern.ch/research/Cytokine_Data_Zandi_et_al_2021.xls.
